# Comparisons of weight change, eating habits and physical activity between women in Northern Sweden and Rural New York State- results from a longitudinal study

**DOI:** 10.1186/s12937-015-0078-0

**Published:** 2015-08-29

**Authors:** Kristina Lindvall, Paul Jenkins, Melissa Scribani, Maria Emmelin, Christel Larsson, Margareta Norberg, Lars Weinehall

**Affiliations:** 1 Department of Public Health and Clinical Medicine, Epidemiology and Global Health Unit, Umeå University, S-901 87 Umeå, Sweden; 2 Umeå Centre for Global Health Research, Department of Public Health and Clinical Medicine, Umeå University, S-901 87 Umeå, Sweden; 3 Bassett Healthcare Network Research Institute, One Atwell Road, Cooperstown, NY 13326 USA; 4 Department of Clinical Sciences, Social Medicine and Global Health, Lund University, Jan Waldenströmsgatan 35, S- 205 02 Malmö, Sweden; 5 Department of Food and Nutrition and Sport Science, University of Gothenburg, Box 300, S-405 30 Gothenburg, Sweden

## Abstract

**Background:**

Previous research has focused exclusively on weight loss or weight maintenance following weight loss, i.e. secondary weight maintenance (SWM). The long-term results of SWM have been modest, suggesting that preventing initial weight gain among normal weight or overweight individuals, i.e. primary weight maintenance (PWM), may be more successful. The aim of this study was to compare the pattern of weight change between Swedish and US women and to contrast eating and physical activity between the two countries.

**Methods:**

A questionnaire of attitudes, strategies and behaviours regarding physical activity, food habits, body image and demands to maintain weight was mailed to 4021 Swedish and 3199 US individuals. Subjects had weight measurements taken 10 years apart in the Västerbotten Intervention Programme in northern Sweden, and self-reported weight as part of the Upstate Health and Wellness Study in Upstate New York. The mean 10-year percent weight change, and weight change in kilograms, were calculated between the two countries for nine female age (30, 40, 50 years at baseline) by BMI (20–25, 25–30, 30–35) groups. For the Swedish/US pair showing the largest differences in these two endpoints, analysis of variance, correlations and chi-square tests identified likely contributors to the observed differences in weight change.

**Results:**

For all subgroups combined, the mean percent weight changes for Swedish women and US women were 4.9 % (SD = 5.8) and 9.1 % (SD = 13.7) respectively (*p*<0.001). Differences in 10 year weight change between the two countries were largest among normal weight 30 year olds. Eight variables were identified as likely contributors to this difference. A significantly higher proportion of Swedish women selected the healthy alternatives for these eight variables. Percent weight change varied considerably over healthy versus unhealthy response levels in the US, but not in Sweden.

**Conclusions:**

The prevalence of obesity among the Swedish women did not progress as rapidly as among the US. The greatest weight gain occurred predominantly among the 30 year old groups. The Swedish women tended to select healthier alternatives than their US counterparts, and women in the US appeared to be more vulnerable to the effects of unhealthy habits than women in Sweden.

## Background

Globally, the prevalence of obesity has nearly doubled over the last three decades [1] and it is one of the major risk factors contributing to the global burden of disease [2]. Obesity has been shown to be associated with diabetes, cardiovascular disease (CVD), several cancers and osteoarthritis [2–4]. In addition, it has been associated with chronic pain. Mental conditions that have been associated with obesity include depression and anxiety [5]. There are also negative psychosocial and/or psychological consequences of obesity due to negative portrayal in the media [6], being discriminated against at the workplace [7, 8] and inequities in health care [9].

This study is based in northern Sweden and the Northeast United States. Self-reported data from Sweden (year 2010–2011) indicate a prevalence of overweight and obesity of 42.0 and 11.8 % respectively among men and 28.4 and 10.5 % respectively among women [10]. Comparable data from the US Behavioral Risk Factor Surveillance System (BRFSS) (self-reported data from 2011), indicate the prevalence of overweight and obesity to be 41.6 and 28.3 % respectively among men and 29.2 and 27.4 % respectively among women [11].

Some short-term weight loss programs among adults have shown significant results [12, 13]. However, this is typically followed by an episode of weight gain, which results in little or no long-term weight loss [14–16]. A shift in focus from weight loss to weight maintenance has therefore been suggested [17–19]. Accordingly, the WHO has recommended *prevention* of weight gain and *promotion* of weight maintenance as the first two basic steps in the effective control of obesity [20]. Despite this, while many investigators and public health advocacy groups have tried to develop strategies to assist in weight reduction (or weight maintenance following weight reduction), fewer have considered how to provide support for long-term weight maintenance [19].

Weight maintenance may be dichotomized into two subcategories: Primary weight maintenance (PWM) and secondary weight maintenance (SWM) [21, 22]. SWM, which has been more commonly studied, refers to maintaining a reduced weight following weight loss. In the majority of studies that fall into the SWM category, the participants do not fare well at maintaining their reduced weights [12–16]. It is possible that this overall result is partially caused by their antecedent weight gain. As a result, it can be hypothesized that a subject who has not experienced this antecedent weight gain may have fewer barriers to maintaining his/her weight.

It is this hypothesis that has led to the development of the concept of PWM, which is the prevention of weight gain among normal weight and overweight individuals. An additional benefit of the PWM-focus is that it enables subjects to prevent initial weight gain, which in turn spares them from the challenge of trying to lose weight.

PWM has received very little attention in the literature. Some initial steps towards developing the concept have been taken in northern Sweden [21–23]. The first of these studies used regression analyses to identify subject characteristics that tended to be predictive of 10-year weight change [23]. In the second study, qualitative in-depth interviews were conducted to explore the attitudes, behaviours and strategies of importance for PWM [22]. In a third study, Analysis of variance (ANOVA), correlation, and linear regression analyses were conducted to identify attitudes, strategies, and behaviours that are predictive of PWM in different age, sex and BMI subgroups in Sweden [21]. The next step, and the focus of the current study, is to contrast these attitudes, strategies and behaviours between two countries that have experienced a different pattern of weight change in the last decades.

The present study makes cross country comparisons between females living in Northern Sweden and rural New York State in the US. There is a strong emphasis on female weight loss in western media and society and a corresponding high demand on women to lose or not gain weight in the industrialized west [24–26]. This focus on weight loss may have obscured the factors that contribute to healthy weight maintenance.

The aims of the present study are:

To compare the pattern of weight change between Swedish and US women within certain demographic subgroups.

To contrast eating and exercise habits between the two countries that may explain the differences in weight change.

## Subjects and methods

### Setting

The study was conducted in 2009 in one Swedish and one US setting. Both settings have long-term ongoing health surveys that will be explained further below.

The Swedish setting is Västerbotten County, which is located in the northern part of the country. The population of the county is approximately 260,000, with about 45 % living in the largest city [27]. The remainder of the population is situated in two smaller cities and the surrounding countryside. The US setting covers a 7-county region of upstate New York [28]. The population of this region is approximately 780,000 with the vast majority living in rural areas.

### The two health surveys

In order to be eligible, the Swedish subjects must have participated at least twice in the Västerbotten Intervention Programme (VIP), which was initiated in 1985 to reduce risk factors for diabetes and CVD [29]. The intervention was integrated into routine health care delivery, with all inhabitants in the county of Västerbotten being invited to participate as of their 30^th^, 40^th^, 50^th^ and 60^th^ birthday. VIP visits are performed at the subject’s primary health centre and are focused on risk factors for CVD. The visit includes height and weight measurement, blood pressure measurement, an oral glucose tolerance test, and blood lipid analysis. Participants also answer a questionnaire covering the following areas: socioeconomic and psychosocial conditions, health-related quality of life, self-rated health, personal health history and family history of CVD and diabetes, social network and support, working conditions, physical activity, alcohol consumption, tobacco consumption, eating habits and a food frequency questionnaire.

Consecutive cross-sectional VIP-data (based on measured weights and heights) showed that the prevalence of overweight and obesity had increased among women from 32.2 and 12.7 % as of 1995 to 33.3 and 16.5 % as of 2007 [30]. For men the prevalence had increased from 47.2 and 10.0 % (overweight vs. obese) as of 1995 to 49.4 and 17.3 % as of 2007.

All US respondents had participated in a longitudinal health study that began in 1989, originally described as the Bassett Health Census, and later referred to as the Upstate Health and Wellness Study [28]. The Upstate Health and Wellness Study did not include any health examinations. The study instead surveyed self-reported chronic disease (including self-reported data on high cholesterol, hypertension, heart disease and diabetes), BMI, and positive and negative behaviours related to chronic disease. A follow-up study conducted in 1999 measured changes in these self-reported parameters over the 10-year period [31, 32]. From 1989 to 1999, the prevalence of self-reported BMI ≥25 in males increased from 53.8 to 63.3 %. In females, an increase from 36.6 to 47.4 % was seen over the same period.

### Inclusion and exclusion criteria

The present study focuses on northern Swedish and US women living in rural New York State, however, the inclusion criteria were dictated by the aims of a previous study [21] including both women and men. The inclusion criteria differed slightly between the two countries. In Sweden, the respondents needed a baseline VIP-measured weight between 1994 and 1998, and a second weight measured 10 years later (2004–2008). As previously stated, the respondents were invited to the VIP the year they turned 30, 40 and 50. However, due to some variations in the timing of the invitation the respondent could be between 29 and 31 (if invited the year they turn 30) by the time of the examination. This meant that the respondents were between 29 and 51 years old as of 1994–1998 and 39–61 ten years later. Thus, the elapsed time between the second weight measurement and the administration of the 2009 questionnaire did not exceed 5 years.

In the US, respondents needed to be between 18 and 55 years of age at the time of the 1999 survey and to have self-reported their height and weight on that survey. In order to maximize comparability between the data from the two countries, a study was performed in the US to derive an equation to correct these self-reported BMIs to estimated measured values [33]. The US data that are discussed below are all corrected using the formulas derived from this study.

In addition, participants were selected on the basis of their baseline BMI with a lower limit of BMI equal to 20 kg/m^2^. This lower limit was set in order to provide three equally wide baseline BMI strata: normal weight (20–25 kg/m^2^), overweight (25–30 kg/m^2^) and obese (30–35 kg/m^2^).

In addition to the stratification by BMI-groups, subjects were also stratified based on sex and baseline age (30, 40 and 50). For the Swedish subjects, the age ranges within these three strata were 29 to 31, 39 to 41, and 49 to 51 respectively. The age ranges within these strata for the US subjects were wider (18 to 35, 36 to 45, and 46 to 55).

The available sample size, which was also based on the aims of the previous study [21], required 150 respondents in each of the age, sex and BMI strata.

Finally, there were two questions in the questionnaire (described in the following section) where the women indicated their body size, at both baseline and 10-year follow-up, using a nine point scale displaying illustrations of body sizes. Responses from women who stated that they were pregnant at either of these two time points were excluded from the study.

### The questionnaire

This study was based on a questionnaire that was constructed using results and hypotheses developed from previous qualitative [22] and quantitative studies [23]. It also included five VIP questionnaire items and seven derived from qualitative interviews conducted in the US setting. A pilot test of the questionnaire was performed on 35 individuals with a similar demographic profile as the study participants. The final questionnaire included 31 questions (many with numerous sub-questions). It assessed attitudes, strategies and behaviours regarding physical activity, food habits, tobacco use, body image and perceived demands to maintain weight. Subjects were asked to answer these questions using five-level Likert scales.

In late May of 2009, an initial mail-out was performed in Sweden that included the questionnaire and study information. This was sent to the entire study population including both women and men. A reminder post card was sent 1 week later informing the recipients that they could still complete and return the questionnaire. A final request to complete the survey was sent to any recipient who had not completed the survey by mid-June. No incentives for participating were given.

In the US, the questionnaire, along with a coupon for a free quart of milk, was initially mailed in June of 2009. Those who did not return this initial survey within 4 weeks received a second copy of the questionnaire with a reminder letter to complete it. Subjects that did not respond to this reminder letter within 3 weeks were classified as non-responders. A random sample of 367 non-responders was then selected for mail and telephone conversion that included a $20 incentive.

### Response rate

Initially, 4062 individuals (including both women and men) were selected to take part in the Swedish study. Out of these, 29 were not available to participate due to moving out of the county or passing away. Another twelve did not receive the questionnaire due to a mailing error or an incorrect address. Of the 4021 remaining, 2138 chose to participate, resulting in a response rate of 53 %. Of these, 110 could not be included in the study. This was due to the subjects removing their “id” number from the questionnaire (*n* = 7), refusal to permit linking of data to VIP (*n* = 95) and participation in VIP in 1993 (*n* = 8) (these last eight subjects were excluded so that no subject had more than 5 years between their VIP follow-up and the administration of the survey).

For the US study, 1347 subjects chose to participate after the initial mailings (including both women and men). This resulted in an initial response rate of 42.1 %. A random sample of 367 non-responders was selected for conversion via a combination of mail and telephone follow-up. Of these, 43.6 % were successfully converted. Accounting for the proportion of the study population represented by these two response strata resulted in an overall estimated response rate of 67 %.

For the present paper, data from 2062 female participants from both countries were used. Of these, 51.5 % were Swedish and 48.5 % were from the US.

#### Analysis

For each country, the mean 10-year percent weight change, and weight change in kilograms, were calculated for each of the nine female age (30, 40, 50 years at baseline) by BMI (20–25, 25–30, 30–35) groups.

Ten-year percent weight change was defined as:

$$ \frac{\mathrm{The}\ \mathrm{follow}\ \mathrm{up}\ \mathrm{weight}\ \hbox{-} \mathrm{The}\ \mathrm{baseline}\ \mathrm{weight}}{\mathrm{The}\ \mathrm{baseline}\ \mathrm{weight}}\times 100 $$

For each BMI and age group, the percent weight change and the weight change in kilograms were compared between the countries in order to identify the Swedish-US pair showing the largest differences. This pair was further studied to identify questionnaire variables that related to this difference.

Prior to further examination of this pair, the five-level Likert scale variables from the questionnaire were collapsed into three levels in order to minimize sparseness. Thus, “strongly agree” and “agree” were reclassified into “agree”, “unsure” remained as “unsure”, and “disagree” and “strongly disagree” were reclassified into “disagree”. Likewise, “always” and “often” became “usually”, “sometimes” remained as “sometimes”, and “seldom” and “never” were reclassified as “rarely”. Questions where one response level (for example “agree”) was selected by more than 96 % of the respondents were eliminated from further consideration.

Identification of the US–Swedish pair to be contrasted involved two separate analyses. In the first, the response pattern to each question was contrasted between the two countries. In the second, the pattern of weight change was compared across the response categories of each question, within each country. This was done to test whether or not percent weight change was related to the subject’s responses.

These two analyses involved three separate steps:

1. Two by three (country by response level) Chi-square tests were performed to detect differences in response patterns between the two countries.
2. A one by three ANOVA was used to compare mean percent weight change across the three response levels of each question. In addition, Pearson correlations between each question and 10-year percent weight change were calculated. If the probability for either the ANOVA or the correlation was significant at *p* ≤ .10, the variable was retained for the next step.
3. The results of steps one and two were then summarized in a table for all variables that were deemed to be modifiable by either the individual or society as a whole. This limitation was imposed because the study aimed at identifying variables that could be potential candidates for use in interventions. These modifiable variables were then evaluated based on the following criteria:
  - Significantly related to percent weight change in the Swedish subgroup with a difference in percent weight change between the “agree” and “disagree” categories of more than 2 %. This 2 % restriction was imposed to eliminate spurious findings such as those where the significance was due to a large difference in the “unsure” group.
  - The procedure described in a) was applied for the US subgroup.
  - Having a chi-square result showing a differential response pattern between the two countries.

Variables meeting criteria a and b were retained. Variables meeting either a or b were examined further. If, in the country where the variable was not related to percent weight change this was deemed to be due to a restriction in range, i.e. more than 95 % of all subjects choosing the same response alternative, the variable was retained.

After completing the steps described, another age-BMI subgroup was further analysed. This subgroup was added in an attempt to clarify some of the results that had been observed in the steps of analyses described above. This sub-group had a distinctly different pattern than what was seen in the two other sub-groups. However, since this group was utilized only to clarify the pattern of differences for the reduced variable set, it did not pass through all of the analytic steps described above. Rather, the analyses for this subgroup primarily involved graphing percent weight change as a function of response alternatives.

#### Ethical considerations

The regional Research Ethics Board in Umeå (Dnr 06-071M) approved this study. The participants gave informed consent prior to each VIP visit and also when completing the questionnaire. They were given three options: to not participate, to participate without linkage to VIP data or to participate with linkage to VIP-data. The study was also approved by the Mary Imogene Bassett Institutional Review Board (IRB number 927).

## Results

The number of participants within each subgroup ranged from 26 to 205 (Table 1). The mean ages for all Swedish and US women were 53.1 (SD = 7.1) and 42.4 (SD = 7.5) years respectively. Table 1 also includes baseline characteristics of the respondents such as educational level and self-reported heritage of overweight and obesity.

**Table 1:** Number of participants in Swedish (total *n* = 1061) and US subgroups (total *n* = 1001) as well as socio-demographic and health related characteristics of the respondents within the subgroups

Weight category		Normal weight			Overweight			Obese		
Age group (y)		30	40	50	30	40	50	30	40	50
Swedish women										
Respondents (no. and (%) of total)		149^a^ (7.2)	190 (9.2)	188 (9.1)	114 (5.5)	156 (7.6)	156 (7.6)	32^b^ (1.6)	26 (1.3)	50 (2.4)
Mean (and median) age		34.2 (34.0)	41.5 (41.0)	51.5 (52.0)	34.0 (34.0)	41.5 (41.0)	51.6 (52.0)	34.0 (35.0)	41.4 (41.0)	51.7 (52.0)
Level of education (no. and (%) within subgroup)^c^	Low	9 (6.1)	19 (10.0)	94 (50.0)	13 (11.5)	29 (18.7)	78 (50.0)	2 (6.3)	6 (23.1)	29 (58.0)
	Medium	67 (45.3)	82 (43.2)	27 (14.4)	49 (43.0)	77 (49.7)	27 (17.3)	18 (56.3)	11 (42.3)	10 (20.0)
	High	72 (48.6)	89 (46.8)	67 (35.6)	51 (44.7)	49 (31.6)	50 (32.1)	12 (37.5)	9 (34.6)	11 (22.0)
Proportion (%) of respondents having at least one parent being overweight or obese^d^(n)		37.5 (144)	40.4 (178)	27.5 (174)	68.8 (109)	57.5 (146)	53.5 (144)	65.5 (29)	66.6 (21)	55.0 (40)
US women										
Respondents (no. and (%) of total)		72^a^ (3.5)	205 (9.9)	183 (8.9)	43 (2.1)	117 (5.7)	135 (6.5)	28 (1.4)	94 (4.6)	124 (6.0)
Mean age (and median)		26.6 (28.6)	40.5 (41.1)	47.7 (47.5)	27.5 (29.4)	40.4 (40.7)	48.2 (48.2)	30.4 (31.7)	40.5 (41.2)	47.9 (48.0)
Level of education (no. and (%) within subgroup)^c^	Low	0 (0)	0 (0)	1 (0.6)	1 (2.4)	0 (0)	0 (0)	0 (0)	2 (2.2)	1 (0.8)
	Medium	17 (23.9)	57 (28.2)	41 (23.3)	11 (26.8)	43 (37.1)	38 (28.8)	11 (39.3)	40 (44.0)	43 (34.7)
	High	54 (76.1)	145 (71.8)	134 (76.1)	29 (70.7)	73 (62.9)	94 (71.2)	17 (60.7)	49 (53.8)	80 (64.5)
Proportion (%) of respondents having at least one parent being overweight or obese^d^(n)		40.3 (72)	44.9 (205)	41.0 (183)	65.1 (43)	59.8 (117)	57.0 (135)	64.2 (28)	78.7 (94)	67.7 (124)

^a^ Groups chosen for the primary comparison

^b^ Group chosen for further clarification

^c^ Level of education was divided into low, medium and high level of education ranging from. In Sweden and in the US, low, medium and high level of education corresponds to the following: Low = Elementary school and middle school (maximum 9 years in school) Medium = High school (maximum 12 years in school) High = College or higher

^d^ According to self-reported values

### Ten year weight change for all subgroups

**Fig. 1:**
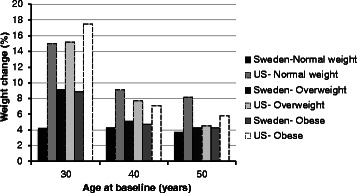
Ten-year percent weight change of Swedish (*n* = 1061) and US (*n* = 1001) women categorized in age and BMI subgroups

**Fig. 2:**
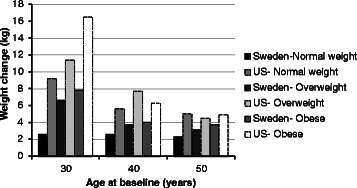
Ten-year weight change in kilograms of Swedish (*n* = 1061 and US (*n* = 1001) women categorized in age and BMI subgroups

For all subgroups combined, the mean percent weight changes during the 10-year period for Swedish women and US women were 4.9 % (SD = 5.8) and 9.1 % (SD = 13.7) respectively (p for *t*-test˂0.001). The median percent weight change over the same period was 4.2 and 7.6 % for the Swedish and US women, respectively. For the Swedish women, the mean weight changes in kilograms was 3.5 (SD = 4.2) while for the US women it was 6.4 (SD = 10.8) (p for *t*-test < 0.001). The median weight change in kilograms was 3.0 for the Swedish women and 4.8 for the US. For the US women, the largest weight change occurred among the 30 year olds for all three BMI strata (Figs. 1 and 2). For the Swedish women, the largest weight change was seen among overweight and obese 30 year old women.

The pair of age and BMI matched subgroups demonstrating the largest difference in 10-year weight change between the countries was normal weight 30 year olds (Figs. 1 and 2). For this Swedish subgroup, the 10-year weight change was 4.2 % (SD = 4.7) while for the US subgroup it was 15.0 % (SD = 16.6) (Fig. 1). The median 10-year percent weight change was 4.1 % and 12.5 for the Swedish and the US subgroup, respectively. The 10-year change in kilograms was 2.6 kg (SD = 2.9) for the Swedish subgroup and 9.2 kg (SD = 10.2) for the US (Fig. 2). The median weight change in kilograms was 3.0 for the Swedish subgroup and 8.0 for the US subgroup.

### Differences in response patterns and variable relationships between the two 30 year old normal weight subgroups

In the Swedish subgroup of 30 year old women, 15 (11.6 %) of the 129 survey variables were significantly related to percent weight change by either ANOVA and/or correlation (step 1). In the US, 42 (29.5 %) of the 129 variables were significant (*p*<0.001). Only three of the 129 variables were significant in both the US and Swedish groups. Using steps 1–3 of the analyses, eight variables were identified as likely contributors to the differences in weight change observed between the two subgroups.

### Proportion of respondents in each subgroup “agreeing” with the final eight variables

The number of respondents, within each country, choosing either “agree”, “unsure” or “disagree”, for these final eight variables are shown in Table 2. A significantly higher proportion of the women in the Swedish subgroup stated that they are physically active “because if they are not they begin to miss it”, “to prevent an injury or disease” and/or “to accomplish work or transportation”. There was also a significantly higher proportion of women in the Swedish subgroup who stated that they “exercised weekdays/daily” and “maintained their exercise habits even during vacations”. Conversely, a significantly higher proportion of the US women stated that they “enjoyed eating snack foods”, “rewarded themselves with food” and “exercised less in the winter”.

**Table 2:** Response patterns to the final eight variables for the 30 year old normal weight Swedish and US women

Variable name	Country	Agree	Unsure	Disagree	Total
		n (%)	n (%)	n (%)	
I enjoy eating snack foods	Sweden	21 (14.1)	13 (8.7)	115 (77.2)	149
	US	52 (72.2)	13 (18.1)	7 (9.7)	72
I reward myself with food	Sweden	19 (12.8)	20 (13.6)	109 (73.6)	148
	US	29 (40.3)	10 (13.9)	33 (45.8)	72
I am physically active because if I am not I begin to miss it	Sweden	120 (81.1)	6 (4.0)	22 (14.9)	148
	US	31 (51.7)	7 (11.6)	22 (36.7)	60
I am physically active to prevent an injury or disease	Sweden	123 (83.1)	6 (4.1)	19 (12.8)	148
	US	28 (45.9)	12 (19.7)	21 (34.4)	61
I am physically active to accomplish work or transportation	Sweden	90 (60.4)	14 (9.4)	45 (30.2)	149
	US	7 (11.5)	10 (16.4)	44 (72.1)	61
I exercise weekdays/daily	Sweden	144 (96.6)	3 (2.1)	2 (1.3)	149
	US	44 (61.1)	6 (8.3)	22 (30.6)	72
I maintain my exercise habits even during vacations	Sweden	100 (67.1)	14 (9.4)	35 (23.5)	149
	US	25 (34.7)	7 (9.7)	40 (55.6)	72
I exercise less in the winter	Sweden	45 (30.2)	10 (6.7)	94 (63.1)	149
	US	43 (59.7)	4 (5.6)	25 (34.7)	72

### Weight change across response alternatives for retained variables

The mean percent weight change for each response level of these eight variables is presented in Table 3. In general, there was a tendency for the percent weight change to vary strongly over levels in the US, but not in Sweden. For example, for the US women, there was more than a 10 % difference in weight change for the “agree” versus “disagree” responses for the variables “I enjoy eating snack foods”, “I am physically active to prevent an injury or disease”, “I exercise daily” and “I am physically active to accomplish work or transportation”. The same contrasts for the Swedish subgroup, showed a maximum difference of one percent. In Sweden, these differences only reached statistical significance for one of these final eight variables (I enjoy eating snack foods). In contrast, for the US subgroup, significance was seen for all eight. The same general pattern was observed for weight change in kilograms.

**Table 3:** Mean percent weight change contrasted across response levels for the final eight variables of Swedish (*n* = 149) and US (*n* = 72) normal weight 30 year old women. The alternatives that were deemed as healthy are in bold font

Variable name	Country	Mean percent weight change for response alternative	Mean percent weight change for response alternative	Mean percent weight change for response alternative
		Agree (mean % weight change)	Unsure (mean % weight change)	Disagree (mean % weight change)
I enjoy eating snack foods	Sweden	3.1	6.9	**4.1**
	US	14.7	9.8	**27.1**
I reward myself with food	Sweden	3.6	3.6	**4.4**
	US	20.5	10.2	**11.5**
I am physically active because if I am not I begin to miss it	Sweden	**4.3**	3.8	3.7
	US	**9.8**	16.5	20.3
I am physically active to prevent an injury or disease	Sweden	**4.2**	3.9	4.1
	US	**9.6**	14.8	21.0
I am physically active to accomplish work or transportation	Sweden	**4.3**	2.6	4.4
	US	**5.8**	9.3	17.1
I exercise weekdays/daily	Sweden	**4.2**	1.1	3.8
	US	**11.5**	7.7	23.9
I maintain my exercise habits even during vacations	Sweden	**4.3**	4.0	3.9
	US	**5.7**	25.4	19.0
I exercise less in the winter	Sweden	4.5	5.3	**3.9**
	US	20.5	4.3	**7.3**

These eight variables were further divided into healthy versus unhealthy choices. For example, agreeing with the statement “I enjoy eating snack foods” was considered to be an unhealthy alternative while agreeing with “I exercise weekdays/daily was considered to be a healthy alternative. The healthy alternatives are bolded in Table 3. For Swedish women the differences for those choosing the unhealthy versus the healthy alternatives was negligible (0.24 kg and 0.32 %). In contrast, the unhealthy alternatives for US women were associated with 5.12 kg or 8.59 % greater weight gain than for the healthy alternatives. There were also noticeable differences when comparing weight change between countries for the same response alternatives. For the healthy alternative, the average US women gained 4.3 kg more than the average Swedish woman (data not shown). In contrast, for the unhealthy alternative the average US woman 9.7 kg more than the average Swedish woman.

### Comparisons of normal weight 30 year old women with Swedish obese 30 year old women

Obese 30 year old Swedish women were selected in order to provide a contrast to the results from the normal weight 30 year old women from both countries. These obese 30 year old women were one of the Swedish subgroups with the highest 10-year weight change (Figs. 1 and 2) in terms of both percent (8.9 % SD = 5.7) and kilograms (7.8 SD = 4.9). Thus, this provided a comparison between the group that had gained the least and the one that had gained the most within Sweden, and also provided a contrast between two groups with similar weight gain between Sweden and the US.

**Fig. 3:**
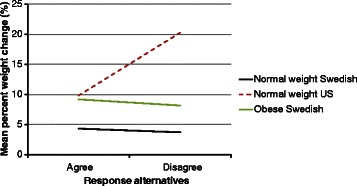
Mean percent weight change between the response alternatives “agree” and “disagree” to the question “I am physically active because if I am not I begin to miss it” among normal weight (Sweden, *n* = 148 and US, *n* = 60) and obese (Sweden, *n* = 31) 30 year old women

In almost all cases, the patterns seen for these obese Swedish women were distinctly different from the normal weight subgroups of either country. The tendency for only small differences in percent weight change between the healthy and the unhealthy response levels in this group was similar to that observed in the normal weight Swedish women. However, the levels of percent weight change at each response level were two to three times as great, resulting in the vertical displacement of the line shown in Fig. 3. This general pattern was seen for six out of the eight final variables considered. These six included “I enjoy eating snack foods”, “I reward myself with food”, “I am physically active because if I am not I begin to miss it”, “I am physically active to accomplish work or transportation”, “I maintain my exercise habits even during vacations” and “I exercise less in the winter”.

It should be noted that contrasts of percent weight change between normal weight and obese groups is problematic due to the large disparity in baseline weight which is the denominator for this endpoint.

**Fig. 4:**
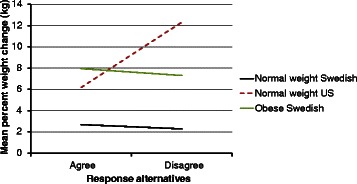
Mean weight change in kilograms between the response alternatives “agree” and “disagree” to the question “I am physically active because if I am not I begin to miss it” among normal weight (Sweden and US) and obese (Sweden, *n* = 31) 30 year old women

A similar pattern was observed when considering weight change in kilograms (Fig. 4) with the exception of three of the eight variables (Fig. 6). These were “I am physically active to accomplish work or transportation”, “I am physically active to prevent an injury or disease” and “I exercise weekdays/daily”. In these three cases, the slope of the line for the obese Swedish women was decidedly steeper than for the normal weight Swedish women. These steeper slopes were the result of a distinctively greater weight gain for the unhealthy versus the healthy alternative.

**Fig. 5:**
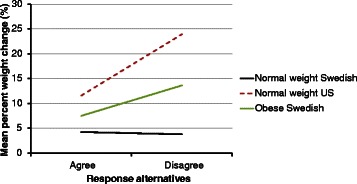
Mean percent weight change between the response alternatives “agree” and “disagree” to the question “I exercise weekdays/daily” among normal weight (Sweden, *n* = 149 and US, *n* = 72) and obese (Sweden, *n* = 31) 30 year old women

The contrast between the normal weight 30 year old US women and the obese 30 year old Swedish women for percent weight change produced what could be characterized as a disordinal interaction. Specifically, for the healthy alternative, the US group showed lesser or equal percent weight gain (Fig. 5) while, for the unhealthy alternative, they showed dramatically greater percent weight change.

**Fig. 6:**
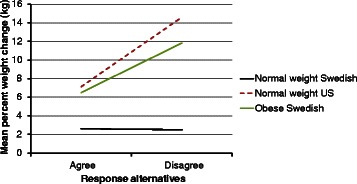
Mean weight change in kilograms between the response alternatives “agree” and “disagree” to the question “I exercise weekdays/daily” among normal weight (Sweden, *n* = 149 and US, *n* = 72) and obese (Sweden, *n* = 31) 30 year old women

For the simpler contrast between these two groups using kilograms, three patterns were observed. For the three variables identified above: “I am physically active to accomplish work or transportation”, “I exercise weekdays/daily” and “I am physically active to prevent an injury or disease”, a similar pattern of much greater weight gain for the unhealthy versus healthy alternative was observed for both subgroups (Fig. 6). For four of the remaining five variables, there was a greater weight gain for the unhealthy versus the healthy alternative for the US subgroup. In contrast, little or no difference was observed between the healthy and unhealthy alternative in the obese Swedish group (Fig. 4). A paradoxical result was observed in the US subgroup for one variable, “I enjoy eating snack foods”, where the greater weight gain was observed for the healthy versus the unhealthy alternative. There was little or no difference in weight gain (kg) between the healthy and unhealthy alternative for this variable in the obese Swedish group.

## Discussion

A previous study conducted between 1989 and 1999 in these two settings showed that the prevalence of obesity rose from 9.6 to 18.4 % in Sweden and from 21.3 to 32.3 % in the US [34]. Further, cumulative distribution curves showed that the Swedish BMI distribution during 1999 was nearly identical to the US distribution during 1989. In addition, the authors observed that Sweden’s obesity increase had a progression similar to that of the US, implying that by 2009, the prevalence of obesity in the Swedish setting might equal the 1999 US level. Since this study was conducted in 2009 these data provide an opportunity to check the validity of this projection.

Taken across all subgroups combined, the weight change in this study, in both percent and kilograms, was almost twice as large for women in the US as in Sweden (4.9 versus 9.1 % and 3.5 versus 6.4 kg). This result indicates that the Swedish obesity development may actually have a slower progression than in the US. This was also supported by a study presenting data from the Swedish setting showing that the prevalence of obesity in Sweden by the year 2007 was 17.3 % among men and 16.5 % among women [30] and not 32.3 %, as projected in that prior study [34].

The largest percent weight change in the US women occurred among 30 year olds regardless of baseline BMI. In contrast, in the Swedish cohort, this trend was observed in the overweight and obese 30 year old women but not in the normal weight. Other studies from Denmark and the US have also found large weight gain to occur in younger age groups [35, 36]. Since the greatest weight change was seen among the youngest US women (regardless of baseline BMI), a study focusing on this group and their barriers and facilitators for PWM would be of great interest.

The three steps of analyses yielded eight variables which strongly emphasized differences both between the two countries and between the BMI groups. When only comparing differences in responses to these eight variables, the answers to what lies behind the large differences in weight gain for normal weight 30 year old women may seem rather straight forward. The Swedish women stated having more of the “healthy” behaviours (such as exercising daily and maintaining eating habits during vacations) with the US women stated having more of the “unhealthy” ones (such as to enjoy eating snack foods and rewarding themselves with food). This would imply that the solution is simply to have the US women start behaving as the Swedish women. This notion however, is complicated by the fact that for the normal weight Swedish women the percent weight gain is relatively insensitive to the choice of healthy versus unhealthy alternatives. In sharp contrast, the US women appear to be profoundly affected by these choices. This relative insensitivity of the Swedish women to the healthy versus unhealthy alternative requires explanation and may be one of the key elements relating to the phenomena of weight gain and weight maintenance.

What might explain the differences in the impact of these variables on weight gain? One possibility may be the different environments surrounding the individuals in these two countries. The US setting may be more obesogenic, wherein it is more important to follow these weight maintaining behaviours. In contrast, the Swedish setting may be more conducive to physical activity, making the weight maintaining behaviours somewhat less important.

For example, in the Swedish setting there are more sidewalks than in the US setting, and these sidewalks are much wider. It may also be of importance that a litre of gas is twice as expensive in Sweden as in the US (Sweden 14 SEK/l ≈ 8.29 dollar/gal vs. US 3.77 dollar/gal ≈ 6.37SEK/l) [37, 38]. It may also be that the Swedish women have a higher Nonexercise Activity Thermogenesis (NEAT) compared to US women. NEAT has been described as “the energy expended for everything that is not sleeping, eating, or sports-like exercise” [39]. It has been proposed to be an important contributing factor to both prevention of weight gain and promotion of weight loss [39–41]. This would be interesting to examine in future studies. In addition, a qualitative study aiming at culturally defining what constitutes “physical activity” in the two settings would be of great interest.

There is also the issue of larger portion sizes in the US. Even though the portion sizes in the Nordic countries have also become larger [42, 43] the US portion sizes have still been shown to be larger compared to those in Europe [43]. One American study obtained information regarding current portion sizes from food manufacturers and via direct weighing [44]. Information on past portion sizes was gathered from food manufacturers and contemporary publications. This study showed portion sizes began increasing in the 1970’s, rose sharply in the 1980’s, and have continued increasing in parallel with increasing body weights. It further showed that market place portion sizes have exceeded federal standards on portion sizes of the US Department of Agriculture and the US Food and Drug Administration.

It is also possible that the Swedish participants have been influenced by the VIP intervention [29]. The extent to which this programme has affected the entire population in terms of PWM, healthy eating and physical activity habits requires further study. The Upstate Health and Wellness Study does not include an intervention. Furthermore, the entire adult population of Västerbotten has been included in an ongoing community intervention to reduce CVD risk since the early 1990s.

The findings of this study indicate that the next step in the field of weight maintenance would be to study environmental factors of importance for PWM. The finding that women in the US seem to be more vulnerable to the effects of unhealthy habits than Swedish women could not be addressed in-depth within this study. Environmental factors that affect PWM may exist on different levels, including the individual, interpersonal, organizational, community, and public policy levels, as cited by the ecological perspective [45, 46]. The ecological perspective further emphasizes the interaction between, and interdependence of, factors across all levels of a health problem. It highlights people’s interactions with their physical and sociocultural environments [46]. A future study of environmental factors of importance PWM using the ecological perspective in the design of the study could be of great interest.

### Methodological considerations

The large number of univariate statistical tests that were used resulted in a very high experiment wide type 1 error probability. Despite this, a correction, such as Bonferroni’s, was not used to adjust this error rate. This was not done because the intention of the study was to identify the maximum number of variables possible that could be used in the planning of a future intervention. It should also be acknowledged that this was an observational study, which precludes the possibility of establishing causality. In addition, some variables would require further study to determine if they are considered as healthy or unhealthy by the participants. Examples of such variables are: “I enjoy eating snack foods” and “I reward myself with food”.

Another limitation of the study is that the low end of the age range in the US (18) was lower than the one in Sweden (29). However, a sub-analysis that excluded all US women under the age of 29 did not alter the conclusion of the study. Another limitation is that the time between first and the second measured weight is ten years. This could result in recall bias for certain variables. In addition, this study was limited in its inability to exclude subjects that may be afflicted with certain diseases that might alter diet, physical activity, and/or the time course of weight change, since data on comorbid conditions were not available for both countries.

The use of two outcome variables (percent weight change and weight change in kilograms) was essential for two reasons. First the use of the kilogram outcome facilitated the contrast between the subjects who were obese versus normal weight at baseline, in which circumstance the contrast of percent weight change would be very difficult to interpret. Percent weight gain is useful for comparison of groups with relatively similar baseline characteristics. One benefit of studying 30 year olds is that they are at particularly high risk for weight gain and, as a result, are most likely to benefit from an intervention.

## Conclusion

This study showed that the prevalence of obesity among Swedish women continued to increase. However, it did not keep pace with the increase in the US. Thirty year old women in the US (regardless of baseline BMI) had the largest increases in weight. The same pattern was observed among the 30 year old Swedish women, except for the normal weight group. Where differences in response alternatives were observed between the two countries, the Swedish women were more likely to select the healthy food and exercise habits. Further, women in the US also seemed to be more vulnerable to the effects of unhealthy habits than Swedish women. Additional studies are needed to find the cause of this apparent vulnerability of the US women. These causes may hold one of the keys to slowing the increasing prevalence of obesity, and facilitating primary weight maintenance, in both countries.
